# Breaking through stent overgrowth: stepwise endoscopic removal using electrosurgical techniques

**DOI:** 10.1055/a-2811-5621

**Published:** 2026-03-02

**Authors:** Vincenzo Vadala', Marta Andreozzi, Matteo Colombo, Marco Spadaccini, Alessandro Fugazza, Cesare Hassan, Alessandro Repici

**Affiliations:** 1 9268 Gastroenterology and Digestive Endoscopy Unit, IRCCS Humanitas Research Hospital, Rozzano, Milan, Italy; 2 437807 Department of Biomedical Sciences, Humanitas University, Pieve Emanuele, Milan, Italy

Self-expanding metal stents (SEMSs) represent the gold standard for palliation of malignant esophageal strictures 1 . However, tumor ingrowth and benign hyperplastic tissue reactions may lead to recurrent dysphagia and firm stent embedment, which makes stent removal challenging 2 . In these situations, the stent-in-stent technique is considered the standard approach; nevertheless, it increases costs and is not feasible in patients who have already undergone this strategy 3 . Thermal ablative techniques, such as argon plasma coagulation, have been used to treat hyperplastic tissue, but available data regarding their safety are limited 4 . In complex cases, the use of electrosurgical devices may allow safe and effective stent retrieval 5 .

We report the case of a 75-year-old patient with esophagogastric junction cancer who had previously undergone two SEMS placements using the stent-in-stent technique. Despite a good oncological response to therapy, the patient developed severe recurrent dysphagia ( Video 1 ). Endoscopic evaluation revealed an impassable luminal stenosis caused by tissue overgrowth at the proximal flange. Radial incisions of the hyperplastic tissue were first performed using a needle knife, exposing the stent mesh of the proximal edge and allowing the successful retrieval of the inner stent with rat-tooth forceps. Inspection of the remaining outer stent showed deep embedment of the distal flange in the gastric wall due to exuberant tissue ingrowth. As mechanical traction was unsafe, an ESD knife (ClearCut J type) was used to carefully dissect the hyperplastic tissue, allowing the complete release and extraction of the stent. The patient was discharged on the same day without adverse events. Follow-up endoscopy at 1 week confirmed restored luminal patency.

Combined endoscopic incision and dissection using electrosurgical knives allowed the stepwise removal of double embedded stents, successfully resolving the luminal stricture caused by tissue ingrowth and overgrowth.Video 1

This case demonstrates that severe tissue ingrowth in stent-in-stent scenarios can be safely managed using endoscopic electrosurgical techniques. The combined use of a needle knife and an ESD knife represents a safe and effective strategy for deeply embedded stent removal, avoiding more invasive interventions.

Endoscopy_UCTN_Code_CPL_1AH_2AD

## References

[1] Spaander MCW van der Bogt RD Baron TH Esophageal stenting for benign and malignant disease: European Society of Gastrointestinal Endoscopy (ESGE) Guideline – Update 2021 Endoscopy 2021 53 751 762 10.1055/a-1475-0063 33930932

[2] Ichita C Sasaki A Kawachi J Esophageal stent removal by stent cutting using the endoscopic submucosal dissection technique Endoscopy 2022 54 02 E935 E936 10.1055/a-1882-4724 35835151 PMC9736841

[3] DaVee T Irani S Leggett CL Stent-in-stent technique for removal of embedded partially covered self-expanding metal stents Surg Endosc 2016 30 2332 2341 10.1007/s00464-015-4475-4 26416379

[4] Inoue T Successful argon plasma coagulation treatment for self-expandable metal stent obstruction in colorectal cancer Oxf Med Case Rep 2022 2022 omac103 10.1093/omcr/omac103 PMC9589472 36299665

[5] Guo KY Ma LY Liu ZQ Endoscopic Sub-Stent Dissection for Removal of Long-Term Embedded Esophageal Self-Expanding Metallic Stents J Gastroenterol Hepatol 2025 40 2267 2274 10.1111/jgh.17041 40641384

